# Determinants of cardiovascular disease among type 2 diabetic patients attending diabetic follow-up clinic in Arba Minch general hospital, southern Ethiopia: an unmatched case-control study

**DOI:** 10.1097/MS9.0000000000001951

**Published:** 2024-03-18

**Authors:** Rodas Temesgen Annose, Henok Asefa, Yenealem Gezahagn, Getachew Abebe, Tadiwos Hailu Zewde

**Affiliations:** a School of Medicine; b Department of Anatomy, College of Medicine and Health Science, Arba Minch University, Arba Minch; c Faculty of Public Health; d Faculty of Public Health, College of Medicine and Health Science, Jima University, Jima, Ethiopia

**Keywords:** albuminuria, cardiovascular disease, hypertension, type 2 DM

## Abstract

**Background:**

Cardiovascular disease (CVD) is a major cause of death and disability among patients with type 2 diabetes, especially in low-income and middle-income countries. Type 2 diabetes mellitus (T2DM) patients have a 2–4-fold increased risk of CVD. There is limited data about cardiovascular disease risks and its determinants among T2DM patients in Ethiopia. This study aimed to identify possible predictors of cardiovascular diseases among adults with T2DM in southern Ethiopia.

**Methods:**

A hospital-based unmatched case-control study was conducted at southern Ethiopia Arbaminch Hospital on 196 randomly selected patients with type 2 diabetes on follow-up (98 cases and 98 controls). The authors collected data using a structured interviewer-administered questionnaire, laboratory checklist, and additional document review of T2DM patients. A multivariable binary logistic regression was fitted to identify cardiovascular disease determinants, and the findings were presented using an adjusted odds ratio (AOR) with a 95% CI.

**Result:**

The mean reported age (±SD) of the cases and the controls was 56.3.3 (±8.9) and 52.3 (±9.3) years, respectively. The two identified independent determinants of cardiovascular disease with AOR [95% CI] were hypertension [AOR=4.953, 95% CI (2.47, 9.93) and persistent urine albuminuria [AOR=12.9, 95% CI (3.98, 41.7)].

**Conclusion:**

This study showed that having high blood pressure and persistent urine albuminuria are independent predictors of cardiovascular disease in T2DM patients. The current study setting needs an intervention for mitigating these cardiovascular disease determinants.

## Introduction

Highlights

- Cardiovascular disease (CVD) is a major cause of death and disability among people with diabetes.
- Adults with diabetes have a higher prevalence of CVD than adults without diabetes.
- This study showed that having high blood pressure and persistent urine albuminuria are independent predictors of cardiovascular disease in type 2 diabetes mellitus patients.

Cardiovascular disease (CVD) is a group of diseases of the heart and blood vessels that includes coronary heart disease, cerebrovascular disease, and peripheral arterial disease. Among multiple cardiovascular disease risk factors, diabetes is the most common one^1^.

Type 2 diabetes mellitus (T2DM) patients are highly affected by cardiovascular disease compared with non-diabetes. More than half of all deaths due to T2DM are related to cardiovascular disease, and it was the major cause of disability among this group of people^5^. The risk of cardiovascular disease increases continuously with rising fasting plasma glucose levels, and patients with T2DM are twice as likely to die due to stroke and heart disease compared to patients without T2DM^6^.

In 2021, it is estimated that 537 million people have diabetes, and this number is projected to reach 643 million by 2030 and 783 million by 2045. In addition, 541 million people are estimated to have impaired glucose tolerance in 2021. It is also estimated that over 6.7 million people aged 20–79 will die from diabetes-related causes in 2021^3^. Type 2 diabetes accounts for 90–95% of people with diabetes mellitus^2^.

In low and middle-income countries, as a result of an increase in urbanization and lifestyle changes, the prevalence of diabetes is increasing^4^. In sub-Saharan Africa, diabetes-related cardiovascular complications are considered rare but are increasing and are associated with cardiovascular complications. In one sub-Saharan study, coronary artery disease was found to affect 5–8% of type 2 diabetic patients and cardiomyopathy, up to 50% of all patients. Almost 15% of stroke patients are diabetic, and up to 5% of diabetic patients have a stroke at the time of diagnosis. The prevalence of peripheral vascular disease among T2DM patients also ranges from 4 to 28%^5^.

Worldwide, cardiovascular disease affects 32.2% of all people with T2DM^9^. Cardiovascular disease represents the principal cause of death and morbidity among people with diabetes, especially those with T2DM. Adults with diabetes have 2–4 times increased cardiovascular risk compared with adults without diabetes, and the risk rises with worsening glycemic control. Diabetes has been associated with a 75% increase in mortality rate in adults, and cardiovascular disease accounts for a large part of the excess mortality^5^.

A report from Ethiopia indicated that cardiovascular disease is the second reason for admission in T2DM patients and was the leading cause of death in hospitalized diabetic patients^7,8^. In 2021, there were 1 920 000 cases of diabetes in Ethiopia, which accounts for 1.6% of the Ethiopian population^3^.

Hypertension (HTN), obesity, physical inactivity, smoking, dyslipidemia, age, family history of heart disease, poor glycemic control, persistent albuminuria, retinopathy, neuropathy, oxidative stress, and longer duration of diabetes mellitus were cardiovascular disease risk factors among diabetes patients^9^.

In patients where diabetes mellitus is combined with manifestations of cardiovascular disease, the mortality doubles, with an estimated 12-year reduction in life expectancy^12^. In addition, an increased rate of hospitalization for cardiovascular disease-related causes and decreased quality of life were seen^10^.

In addition, cardiovascular disease in T2DM patients adds a significant economic burden at the individual patient and the population level. It contributed to 20–49% of the direct costs of treating T2DM^14^.

Concerning the growing burden of diabetes and its cardiovascular complications in the developing world, it is crucial to determine the association between other factors independent of DM that predispose diabetic patients to cardiovascular disease. Early identification and stratification would lead to appropriate management of cardiovascular risk factors, reducing morbidity, mortality, and cost expenditure due to the treatment of cardiovascular diseases in this population.

In low-income countries, like Ethiopia, non-communicable diseases are responsible for over 1.8 million deaths per year. Age-adjusted cardiovascular disease-related mortality in the region is high^1^ There is limited research related to cardiovascular disease and T2DM in the current research setting as well as in Ethiopia. Most of the literature so far looked into the prevalence of cardiovascular disease and related risk factors^7,15^. Different studies in different research setups identified risk factors that predispose T2DM patients to cardiovascular disease and a range of prevalence of cardiovascular disease among T2DM patients. Yet, we have limited data on how strongly these identified risk factors are associated with cardiovascular disease outcomes in T2DM patients in our population. This study seeks to identify determinants of cardiovascular disease among T2DM.

## Materials and methods

### Study design, setting, and sampling

A facility-based unmatched case-control study was conducted from 15 May 2022, to 15 June 2022, in Gamo zone, Gamo zone is located 434 km south of Addis Ababa, the capital city of Ethiopia. According to the most recent Ethiopian census, it has a total population of 1 ,852 ,000. One general and three primary hospitals provide curative, preventive, and rehabilitative services for the population in the catchment area. This work has been reported in line with the STROCSS criteria^26^.

### The population of the study

#### Source population

Cases: All diabetic patients in the follow-up clinic with cardiovascular disease.

Controls: All diabetic patients in the follow-up clinic without cardiovascular disease.

#### Study population

Cases: Selected and eligible type 2 diabetic patients with cardiovascular disease.

Controls: Selected and eligible Type 2 Diabetic without cardiovascular disease.

### Sampling

The sample size was calculated by using EPI Info software version 7 with the following Parameters: significance=95%; power=80%; Adjusted odds ratio (AOR)=2.41. The Case-to-control ratio was 1:1, and the hypothetical proportion of cases with exposure was 70.9%. The odds ratio was taken from a study conducted in Harar, Eastern Ethiopia, which took HTN as a risk factor for CVD, resulting in the maximum sample size of 196^27^.

Among the variables considered (alcohol consumption, age, presence of micro-vascular complication, and HTN), HTN was selected as a determinant variable for cardiovascular disease since it gave the maximum sample size^27^.

A systematic random sampling technique was used as a sampling method.

### Data collection procedure

Data were collected using structured questionnaires through the interviewer and translated into local languages (Amharic). A separate checklist for laboratory results was linked with the questionnaire using a medical record number (MRN) as an identification code. In addition, a patient document review was conducted to obtain further information. The questionnaire addresses socio-demographic factors, clinical factors, and behavioural factors.

### Measurements

Tools or techniques to assess cardiovascular disease among T2DM Patients (Determinants of cardiovascular disease among type 2 diabetic patients attending diabetic follow-up clinic, Southern Ethiopia: an unmatched case-control study (figshare.com))

To assess heart failure, we used Framingham criteria, previous echocardiography suggestive of any heart failure, and previously diagnosed patients with either of the two criteria and already on treatment. (Presence of any of the three criteria is adequate) (S1). Those with other reasons for Heart failure, like anaemia, cor-pulmonale, and Thyrotoxicosis, were excluded.

To assess for stroke, a ROSSIER score was used (S1). In addition, brain imaging is suggestive of brain infarction/haemorrhage at any time in the past with a reading from a radiologist and previously diagnosed patient with either of the two criteria and already on treatment. (Presence of any of the three criteria is adequate). History of ischaemic heart disease is measured by the previous history of MI/IHD, typical symptoms of MI or Angina equivalent with ECG changes Typical of MI/ACS (ST-segment elevation, ST-segment depression, Q- Q-waves, T wave abnormalities), and Troponin /CKMB increase. Echocardiography evidence of wall motion abnormality suggestive of coronary heart disease, Diagnosed MI/IHD At any time in the past and on treatment, any of a combination of the above criteria can be used (S1).

Diagnosis of peripheral arterial disease is made based on the history of Peripheral arterial disease on treatment or Doppler ultrasound suggestive of PAD at any time in the past (S1).

Blood pressure (BP) was measured using a mercury sphygmomanometer in a sitting position after 10 minutes of rest in both hands on two different occasions, and an average was taken.

For laboratory measurement, blood was taken at the clinic after overnight fasting of 8–10 hours for determination of FBS and lipid panel (total cholesterol, high density lipoprotein cholesterol, and triglyceride). Low density lipoprotein was calculated based on the Freidwald formula. A urine dipstick was done for the presence of albuminuria. A semi-quantitative dipstick test was used to check for albuminuria screening (OneCare medical dipstick urinalysis kit with a detection cutoff value starting at 30 mg/dl). Persistent albuminuria was defined as albuminuria that persists for 3 months. Two urine albumin tests at least 3 months apart were taken for analysis, and the urine dipstick method was used to check the presence of albuminuria in at least two-morning spot samples. A fasting plasma glucose level was determined using a glucometer, and an average was taken from the three most recent records; a mendry chemistry analyzer was used to analyze the lipid panel.

The trained clinical nurse carried out the interview and physical measurements. A physical examination and chart review were performed by a trained medical doctor. A laboratory sample was collected by an experienced nurse working at the diabetic clinic. A senior laboratory technician carried out laboratory analysis. The general practitioner supervised data collection at the diabetic clinic.

### Data processing

Data collectors were trained to ensure data quality. Data were checked for completeness daily, edited, coded, and entered into Epi data version 3.1, and exported to SPSS 25.0 statistical software for analysis. Data cleaning was done for inconsistencies and missing values. Replacing missing values using the imputation method was done to deal with any missing information on covariates. Two-sample *t*-test and Wilcoxon–Mann–Whitney test were used to compare data between groups. Descriptive statistics such as mean, frequency, and percentage were calculated, and data were presented using text and charts. Bivariate analysis was done, and all explanatory variables that had an association with the outcome variable at a *P* value less than 0.25 were included in the multivariable analysis model. Multivariable analysis was employed to determine independent predictors. The odds ratio with its 95% CI was used to decide whether those independent variables included in the multivariable analysis were statistically significant or not.

## Result

### Socio-demographic characteristics

Ninety-eight cases and 98 controls were included in the study. The majority of 54 (55.1%) cases and 55 (56.1%) control were female. The mean reported age (±SD) of the cases and the controls was 56.3.3 (±8.9) and 52.3 (±9.3) years, respectively. 68 (69.3%) cases and 69 (70.4%) controls were less than 65 years in age, 30 (30.7%) cases, and 29 (29.6%) control were greater than 65 years. Sixty-nine (70.4%) cases and 66 (67.3%) were from urban residences, and 91 (92.9%) cases and 90 (91.8%) control were married (Table 1).

**Table 1:** Socio-demographic characteristics of diabetic patients under follow-up in diabetic clinics, southern Ethiopia

	Case	Control	*P*
Variable	*N* (%)	*N* (%)	
Age categorical			
<65 years	68 (69.3)	69 (70.4)	0.876
65 and above years	30 (30.7)	29 (29.6)	
Sex			
Male	44 (44.9)	43 (43.9)	0.886
Female	54 (55.1)	55 (56.1)	
Residence			
Urban	69 (70.4)	66 (67.3)	0.644
Rural	29 (29.6)	32 (32.7)	
Religion			
Muslim	4 (4.1)	6 (6.1)	0.611
Orthodox	50 (51)	54 (55.1)	
Protestant	44 (44.9)	38 (38.8)	
Educational status			
No formal education	28 (28.6)	39 (39.8)	0.362
Primary education (1–8)	30 (30.6)	22 (22.4)	
Secondary school (9–12)	15 (15.3)	15 (15.3)	
College and above	25 (25.5)	22 (22.4)	
Marital status			
Married	91 (92.9)	90 (91.8)	0.788
Other (Single, divorced/widowed	7 (7.1)	8 (8.2)	
Occupational status			
Governmental worker	40 (40.8)	44 (44.9)	0.653
Housewife	24 (24.4)	26 (26.5)	
Other (farmer, merchant, student)	34 (34.7)	28 (28.6)	

### Clinical characteristics of the study participant

One-third of cases, as well as controls, were diagnosed and lived with DM for more than 10 years. Only a minority of the patients, 18 (18.4%) cases and 22 (22.4%) control, had optimal glycemic control (70–130 mg/dl). The majority (>90%) of the cases, as well as the controls, had regular self-glucose monitoring, and 65 (66.7%) cases and 68 (70.8%) of controls had BP in the normal range during data collection (Table 2). A relatively higher number of cases has persistent urine albuminuria 20 (20.4) compared to the control group 3 (3.1). A minority of patients had an impaired renal function test on their most recent visit 8 (8.3) cases and 2 (2) control. Type of cardiovascular disease among cases include Heart failure (55), Hypertensive heart disease (31), stroke (9), and peripheral arterial disease (24). A single patient may have more than one cardiovascular disease at a time.

**Table 2:** Medical history and clinical factors of type 2 diabetic patients under follow-up in the diabetic clinic in southern Ethiopia

	Case	Control	
Variable	*N* (%)	*N* (%)	*P*
Type of medication used			
Insulin	8 (8.2)	4 (4.1)	0.522
Oral hypoglycaemia drug (Metformin, sulfonylureas)	82 (83.7)	87 (88.8)	
Both	8 (8.2)	5 (5.1)	
Duration of DM			
1–3 years	27 (27.8)	38 (38.8)	0.227
4–6 years	30 (30.9)	23 (23.5)	
7–9 years	14 (14.4)	18 (18.4)	
10 and above years	27 (27.8)	19 (19.4)	
Fasting blood glucose level			
Less than 70 mg/dl	—	—	0.478
71–130 mg/dl	18 (18.4)	22 (22.4)	
>131 mg/dl	80 (81.6)	76 (77.5)	
Method for monitoring glycemic control			
Self-monitoring of blood glucose	98 (100)	98 (100)	
History of DM-related complication			
Yes	6 (6.2)	6 (6.2)	0.971
No	92 (93.7)	92 (93.7)	
Having high blood pressure			
Yes	62 (63.3)	29 (29.6)	0.002
No	36 (36.7)	69 (70.4)	
Presence of dyslipidemia on lab			
Yes	71 (73.9)	58 (59.1)	0.05
No	27 (28.10	40 (40.9)	
Persistent urine albuminuria			
Yes	27 (20.5)	4 (4.1)	0.000
No	71 (72.4)	94 (95.9)	
Impaired renal function test			
Yes	12 (12.2)	7 (7.1)	0.151
No	86 (87.7)	91 (92.8)	
Diabetic drug discontinuation			
Yes	7 (7.1)	6 (6.1)	1
No	91 (92.3)	92 (93.9)	

DM-related any complication: Retinopathy, Nephropathy...e.t.c.

High Blood pressure>130/80 mm Hg; mm Hg- millimetre of mercury; DM, diabetes mellitus; mg/dl, milligram per decilitre.

### Behavioural-related characteristics of the study participant

**Figure 1:**
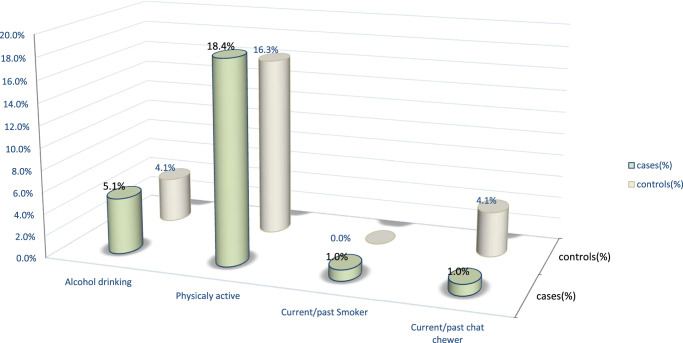
Behaviour-related characteristics of type 2 diabetes patients under follow-up in the diabetic clinic, southern Ethiopia.

Almost all 93 (94.9%) cases and 94 (95.9%) controls have no history of drinking alcohol, and only a minority 18 (18.4) cases and 16 (16.3%) controls were currently involved in physical activities. Almost all patients are currently nonsmokers in both groups presented in Fig. 1.

### Independent predictors of cardiovascular disease

All nine variables with a *P* value less than 0.25 in bivariate analysis were included during multivariable analysis.

After controlling possible confounding factors, having high BP and persistent urine albuminuria was significantly associated with cardiovascular disease. The odds of having persistent urine albuminuria were 13 times higher among cases than controls as compared to no persistent urine albuminuria. [AOR=12.9, 95% CI (3.98, 41.7))]. And the odds of having high BP were five times higher among cases than controls. [AOR=4.953, 95% CI (2.47, 9.93) (Table 3).

**Table 3:** Independent predictors of cardiovascular disease in type two diabetes patients under follow-up in the diabetic clinic, southern Ethiopia

	Case	Control			
Variable	*N* (%)	*N* (%)	COR (95% CI)	AOR (95% CI)	*P*
Having high blood pressure					0.002
Yes	62 (63.3)	29 (29.6)	4.09 (2.25, 7.44)	4.953 (2.47, 9.93)	
No	36 (36.7)	69 (70.4)	1	1	
Persistent urine albuminuria					0.001
Yes	27 (20.5)	4 (4.1)	8.9 (2.9, 26.6)	12.9 (3.98, 41.7)	
No	71 (72.4)	94 (95.9)	1	1	

AOR, adjusted odds ratio; COR, crude odds ratio.

## Discussion

We have performed an unmatched case-control study to determine the determinants of cardiovascular disease in our setting. In this study, HTN and persistent urine microscopic albuminuria were found to have an association with developing cardiovascular disease in T2DM patients.

The odds of having persistent microscopic urine albuminuria were higher among cases than in control. This was consistent with a study done in Danish that reported microscopic urine albuminuria was associated with cardiovascular disease and increased mortality in patients with T2DM^17,18^. In addition, urine albuminuria is associated with endothelial dysfunction which leads to several vascular complications, including cardiovascular disease in type 2 diabetes and the general population^19,20^. In another study, a target-driven treatment of type 2 diabetes with microalbuminuria indicates an increase in lifespan^21^.

Additionally, this study showed that diabetic patients with cardiovascular disease were more likely to have high BP than diabetic patients without cardiovascular disease. This was similar to a study suggesting high BP or HTN as one of the most important risk factors for cardiovascular disease with or without T2DM. Another study showed that 54% of strokes and 47% of coronary heart disease are attributed to high BP^19^. Type 2 diabetes and hypertension are closely related, and HTN is an independent risk factor for cardiovascular disease because of shared risk factors, such as endothelial dysfunction, vascular inflammation, arterial remodelling, atherosclerosis, dyslipidemia, and obesity. There is also an overlap in the cardiovascular complications of diabetes and hypertension. Common mechanisms, such as upregulation of the renin-angiotensin-aldosterone system, oxidative stress, inflammation, and immune system activation, likely contribute to the close relationship between diabetes and hypertension. When both happen to be together, it markedly increases the risk of cardiovascular disease^11,20^. Reducing high BP in patients with newly diagnosed T2DM results in a decrease in the incidence of diabetes-related vascular diseases and other complications^21^.

Different studies showed that a long duration of DM was associated with increased cardiovascular events^22.^ This was inconsistent with this study, where a long duration of DM was not found to be an independent predictor of cardiovascular disease.

The majority of patients (both case and control) in this study were shown to have sub-optimal optimal glycemic control as compared to a recommendation from the American diabetic association target of (70–130 mg/dl)^1^. Despite having sub-optimal glycemic control, poor glycemic control was not shown to be associated with increased odds of cardiovascular disease, while some studies show poor glycemic control is associated with increased cardiovascular events^23–25^. This may be due to the relatively smaller sample size in this study.

## Conclusion

Cardiovascular disease is the major cause of morbidity and mortality in T2DM patients. The determinants for cardiovascular disease among type two diabetic patients in the study settings are persistent urine microscopic albuminuria and elevated blood pressure. Microalbuminuria is an early marker of vascular endothelial dysfunction in patients with diabetes mellitus. Persistent urine albuminuria can be used as a marker and predictor of risk for cardiovascular disease among T2DM patients.

Our study fills the gap in the literature, will help in improving diabetic patient care in the current research setting and serve as input for researchers who will pursue this topic. In addition, this study will contribute to policymakers and programmers' efforts to control and prevent the occurrence of CVD in diabetic patients in Ethiopia.

### Recommendation

The occurrence of cardiovascular disease as a complication of T2DM increases mortality and morbidity and affects the quality of life of patients. Therefore, early identification of modifiable risk factors that can prevent and delay cardiovascular disease-related events is very important. In addition, applying regular screening tools to identify modifiable risk factors is important.

Diabetic follow-up clinics have to do annual screening of patients with T2DM for urine albuminuria, and blood pressure has to be measured at each visit. In those found to have persistent urine microscopic albuminuria, physicians have to initiate angiotensin-converting enzyme inhibitors (ACEI) or Angiotensin receptor blockers (ARB), which are known to reduce urine albuminuria, therefore, slow down cardiovascular events as well as diabetic nephropathy.

Physicians and those involved in the care of T2DM patients have to aim for optimal blood pressure targets. Those with hypertension should also be encouraged to have good BP control with a target of less than 130/80 mmHg or less, as suggested by previous guidelines. The patient should be encouraged to follow lifestyle modification in an attempt to achieve target blood pressure. The use of ACEI or ARB drug as a drug of choice for those with high blood pressure (hypertension) will also address both hypertension as well as albuminuria. Hospitals should strengthen the existing regular follow-up, optimal BP control, and annual screening to identify and treat urine albuminuria. Further action research should be done with intervention on identified determinants.

### Limitations of the study

#### Limitation

Due to the nature of the study design, we cannot be sure all cardiovascular disease cases are related to T2DM, and patients with asymptomatic cardiovascular disease may be missed as well. Variables that depend upon a patient’s ability to recall may be subjected to recall bias. Due to a limited number of cardiovascular disease patients with T2DM non-probability sampling method was used to select cases.

## Ethical consideration

The ethical approval letter was obtained from the Institutional Ethical Review Board with reference number IRB/R1030/2022. Written informed consent was obtained from the study participants after informing the purpose of the study.

## Consent

Written informed consent was obtained from the patients for publication and any accompanying images. A copy of the written consent is available for review by the Editor-in-Chief of this journal on request.

## Source of funding

Not applicable.

## Author contribution

R.T developed the proposal, did the analysis and interpretation of the result, and prepared the manuscript. H.A, Y.G, G.A, and T.H contributed to the proposal and design development, assisted during analysis and revised subsequent draft of the manuscript. All authors read and approved the final manuscript.

## Conflicts of interest disclosure

The author(s) declared no potential conflicts of interest concerning the research, authorship, and/or publication of this article.

## Research registration unique identifying number (UIN)

Not applicable.

## Guarantor

Not applicable.

## Data availability

All data are fully available without restriction. All relevant data are included in the manuscript and Supporting Information files.

## Provenance and peer review

Not invited.
